# Vocal universals and geographic variations in the acoustic repertoire of the common bottlenose dolphin

**DOI:** 10.1038/s41598-021-90710-9

**Published:** 2021-06-04

**Authors:** A. R. Luís, L. J. May-Collado, N. Rako-Gospić, T. Gridley, E. Papale, A. Azevedo, M. A. Silva, G. Buscaino, D. Herzing, M. E. dos Santos

**Affiliations:** 1 MARE - Marine and Environmental Sciences Centre, ISPA - Instituto Universitário, Rua Jardim do Tabaco, 34, 1149-041 Lisboa, Portugal; 2 Projecto Delfim - Centro Português de Estudo dos Mamíferos Marinhos, Rua Jardim do Tabaco, 34, 1149-041 Lisboa, Portugal; 3 Department of Biology, University of Vermont, Burlington, VT 05403 USA; 4 Centro de Investigacion en Ciencias del Mar y Limnologia, Universidad de Costa Rica, San Jose, Costa Rica; 5 Blue World Institute of Marine Research and Conservation, Kaštel 24, 51551 Veli Lošinj, Croatia; 6 Centre for Statistics in Ecology, Environment and Conservation, Department of Statistical Sciences, University of Cape Town, C/O Sea Search Research and Conservation NPC, Cape Town, South Africa; 7 Institute for the Study of Antropogenic Impacts and Sustainability in the Marine Environment, National Research Council, Capo Granitola, Via del Mare 3, 91021 Torretta Granitola (TP), Italy; 8 Department of Life Sciences and Systems Biology, University of Torino, Via Accademia Albertina 13, 10123 Torino, Italy; 9 Laboratório de Mamíferos Aquáticos e Bioindicadores Profª Izabel Gurgel (MAQUA), Universidade do Estado do Rio de Janeiro, Rio de Janeiro, Brazil; 10 OKEANOS & IMAR – Instituto do Mar, Universidade dos Açores, 9901-862 Horta, Portugal; 11 Wild Dolphin Project, P.O. Box 8436, Jupiter, FL 33468 USA; 12 Department of Biological Sciences, Florida Atlantic University, Boca Raton, FL 33431 USA

**Keywords:** Animal behaviour, Behavioural ecology, Marine biology

## Abstract

Acoustical geographic variation is common in widely distributed species and it is already described for several taxa, at various scales. In cetaceans, intraspecific variation in acoustic repertoires has been linked to ecological factors, geographical barriers, and social processes. For the common bottlenose dolphin (*Tursiops truncatus*), studies on acoustic variability are scarce, focus on a single signal type—whistles and on the influence of environmental variables. Here, we analyze the acoustic emissions of nine bottlenose dolphin populations across the Atlantic Ocean and the Mediterranean Sea, and identify common signal types and acoustic variants to assess repertoires’ (dis)similarity. Overall, these dolphins present a rich acoustic repertoire, with 24 distinct signal sub-types including: whistles, burst-pulsed sounds, brays and bangs. Acoustic divergence was observed only in social signals, suggesting the relevance of cultural transmission in geographic variation. The repertoire dissimilarity values were remarkably low (from 0.08 to 0.4) and do not reflect the geographic distances among populations. Our findings suggest that acoustic ecology may play an important role in the occurrence of intraspecific variability, as proposed by the ‘environmental adaptation hypothesis’. Further work may clarify the boundaries between neighboring populations, and shed light into vocal learning and cultural transmission in bottlenose dolphin societies.

## Introduction

For species with a wide geographic distribution, variation in behavioural traits (e.g. foraging preferences, hunting strategies, antipredatory displays or acoustic repertoires) is common, and such differences are often used to distinguish populations^1^. Intraspecific acoustic variants, in particular, have been described for several taxa, at various scales. Even among sympatric populations, vocal variations have long been noted in numerous bird species (see^2^), and a few non-human primates (e.g.^3,4^). Variations between neighboring social groups may be considered true dialects, transmitted through learning, and have been well studied in some cetacean species, such as killer whales^5,6^, sperm whales^7,8^ and pilot whales^9^. On a broader scale, acoustic differences among allopatric populations have been reported in numerous species of insects, fish, anurans, birds, terrestrial mammals (e.g.,^10–12^), and also in marine mammals such as Amazon river dolphins, spinner dolphins and harbor seals^13–15^. Both micro and macro-geographic variations in vocal repertoires may be caused by a multiplicity of genetic, social, ecological and historical factors^16,17^, as selective pressures vary in different eco-ethological contexts.

In cetacean societies, vocal signaling is the primary modality of communication^18^ and acoustic variability appears to be widespread. However, the specific causes and immediate functions of such variations still need clarification. Studies on odontocetes’ acoustic divergence point to different pathways: (i) for species with stable kin groups, such as killer whales and sperm whales, variations in acoustical traits have been correlated with genetic structure, but also associated with cultural identity^19,20^; (ii) for other species that live in fission–fusion groups, such as spinner dolphins and bottlenose dolphins, acoustic variations in some signal types have been linked with the variables related to the context of emission—ecological conditions, group size, group composition and activity patterns^21–24^.

Common bottlenose dolphins (*Tursiops truncatus*) inhabit estuaries, coastal regions and open ocean ecosystems, worldwide in tropical and temperate waters, in resident or transient fission–fusion groups that may range from dozens of individuals to mega-pods of thousands^25^. Within their wide geographical distribution, variability in morphological characteristics (e.g. size, color pattern and dorsal fin shape), molecular genetic profiles and habitat use preferences has been documented^26–29^ and, although there are still much needed taxonomical clarifications, two distinct ecotypes—coastal (or inshore) and offshore (or oceanic)—are acknowledged for various locations (see^29^). Moreover, two subspecies are, currently, recognized for the western South Atlantic: *Tursiops truncatus gephyreus* in the coastal waters of southern Brazil, Uruguay and northern Argentina and *Tursiops truncatus truncatus* (of a more offshore habitat preference)^26^.

As cetaceans, common bottlenose dolphins are acoustically specialized animals that present unique cognitive and communicative characteristics^16,30–32^, and their acoustic skills include the ability to modify and produce novel vocalizations as a result of experience (vocal learning) and capability to imitate sound patterns (vocal mimicry)^16^. Their vast acoustic repertoire includes click trains for echolocation^33–35^, narrow-band frequency-modulated whistles for communication, and a wide variety of burst-pulsed sounds, whose specific functions are still a matter of debate^36–39^.

Although the acoustic emissions of bottlenose dolphins are widely documented^40^, studies on geographic variation are scarce, and mostly focused on the influence of environmental factors on whistles’ emission^23,24,41^. Acoustic divergence in bottlenose dolphins has been assessed by comparing whistles’ features or emission rates in different populations, at a local scale^24,41–43^. However, geographic distance, social behavioural patterns and population genetic structure may also play important roles in acoustic geographic variation^44^.

A comparative analysis of the extended acoustic repertoire of common bottlenose dolphins, at a broader scale, may shed light on the species’ vocal flexibility, its social learning mechanisms and cultural transmission in dolphin societies. With that in mind, our goal is to identify and compare the different vocal elements that comprise the extended acoustic repertoire of several *T. truncatus* populations across the Atlantic Ocean and the Mediterranean Sea, and to highlight the expression of shared vocal elements and acoustic variants among allopatric and sympatric populations.

## Results

### Acoustic repertoire

A total of 7048 vocal elements, namely whistles (N = 2526), burst-pulsed sounds (N = 1640), bray series elements (N = 2552) and bangs (N = 330) were selected for analysis (see Table 1), and categorized into 24 signal sub-types (Table S2).

**Table 1:** Data collection and vocal elements, by location.

	Data collection		Vocal elements				
	Sound recordings (mins.)	Sample size	Whistles	Burst-pulsed sounds	Bray series elements	Bangs	TOTAL
Northeast Atlantic, Sado estuary, Portugal	239	156	949	590	1250	131	2920
Mid-North Atlantic, Azores, Portugal	112	17	423	93	510	5	1031
Adriatic Sea, Croatia	293	65	124	54	234	6	418
Central Mediterranean Sea, Sicily Channel, Italy	81	39	362	155	69	7	593
Southeast Atlantic, Namibia	294	108	82	60	3	4	149
Caribean Sea, Bahamas	86	23	196	405	42	16	659
West Central Atlantic, Panama	174	102	165	92	20	139	416
West Central Atlantic, Costa Rica	93	29	157	166	91	21	435
Southwest Atlantic, Brazil	106	38	68	25	333	1	427

The average number of vocal elements recorded at each location was 15.56 ± 2.36.

Repertoire size varied between 13 signal sub-types, for Namibia, Panama, Costa Rica and Brazil, and 19 sub-types for Sado estuary, Portugal.

### Common signal types and acoustic variants

The broad signal-type categories (whistles, burst-pulsed sounds, bray series elements and bangs) were recorded at all sites. However, only seven signal sub-types (sinusoidal and convex whistles, chirps, creaks, squawks, variable rate click trains and bangs) were common across locations, which represent 29% of calls shared between populations. The occurrence of the other 17 signal sub-types diverged between groups, and acoustic variants were especially notorious for bray series elements (see Table 2).

**Table 2:** Differences in signal sub-types occurrence.

	Whistles					Burst-pulsed-sounds		Bray series elements									
	Upsweep	Downsweep	Concave	Constant frequency	Low-freq chirps	S-BP	Moans	Gulp	Grunt	SC-Squeak	VSC-Squeak	D-Squeak	Up-Squeak	LD-Squeak	Un-Squeak	Cc-Squeak	Sin-Squeak
Northeast Atlantic, Sado estuary, Portugal (Repertoire size = 19)	✓	✓	✓	✓		✓	✓	✓	✓	✓	✓	✓			✓		
Mid-North Atlantic, Azores, Portugal (Repertoire size = 17)	✓	✓	✓			✓	✓	✓	✓	✓		✓		✓			
Adriatic Sea, Croatia (Repertoire size = 18)	✓	✓	✓	✓		✓	✓	✓	✓		✓	✓				✓	
Central Mediterranean Sea, Sicily Channel, Italy (Repertoire size = 17)	✓			✓		✓		✓	✓	✓	✓	✓			✓	✓	
Southeast Atlantic, Namibia (Repertoire size = 13)	✓	✓						*	*	✓					✓		
Caribbean Sea, Bahamas (Repertoire size = 17)	✓				✓	✓	✓	✓	✓	✓	✓	✓	✓				
West Central Atlantic, Panama (Repertoire size = 13)		✓			✓		✓			✓	✓						✓
West Central Atlantic, Costa Rica (Repertoire size = 13)	✓		✓			✓			✓	✓		✓					
Southwest Atlantic, Brazil (Repertoire size = 13)	✓					✓		✓	✓	✓	✓						

✓Present in the data collection.

*Not present in the data collection but reported in previous publications.

While a total of eight sub-types of squeaks were detected, only two to five variants were recorded at each location. The most uncommon sub-types were Up-squeak, LD-Squeak and Sin-Squeak present only in Bahamas, Azores and Panama samples, respectively. On the opposite, SC-squeaks were not observed in Adriatic Sea, gulps were not recorded in Panama or Costa Rica, and grunts were absent in Panama. Within burst-pulsed sounds category, S-BP sub-type was not sampled in Namibia or Costa Rica. Whistles sub-types recorded at each location were also variable: upsweeps were not recorded in Panama, constant frequency whistles were present only in the Mediterranean (Sicily Channel and Adriatic Sea) and in Sado estuary, whereas downsweeps and concave whistles were absent in four locations.

### Repertoire (dis)similarity

**Figure 1:**
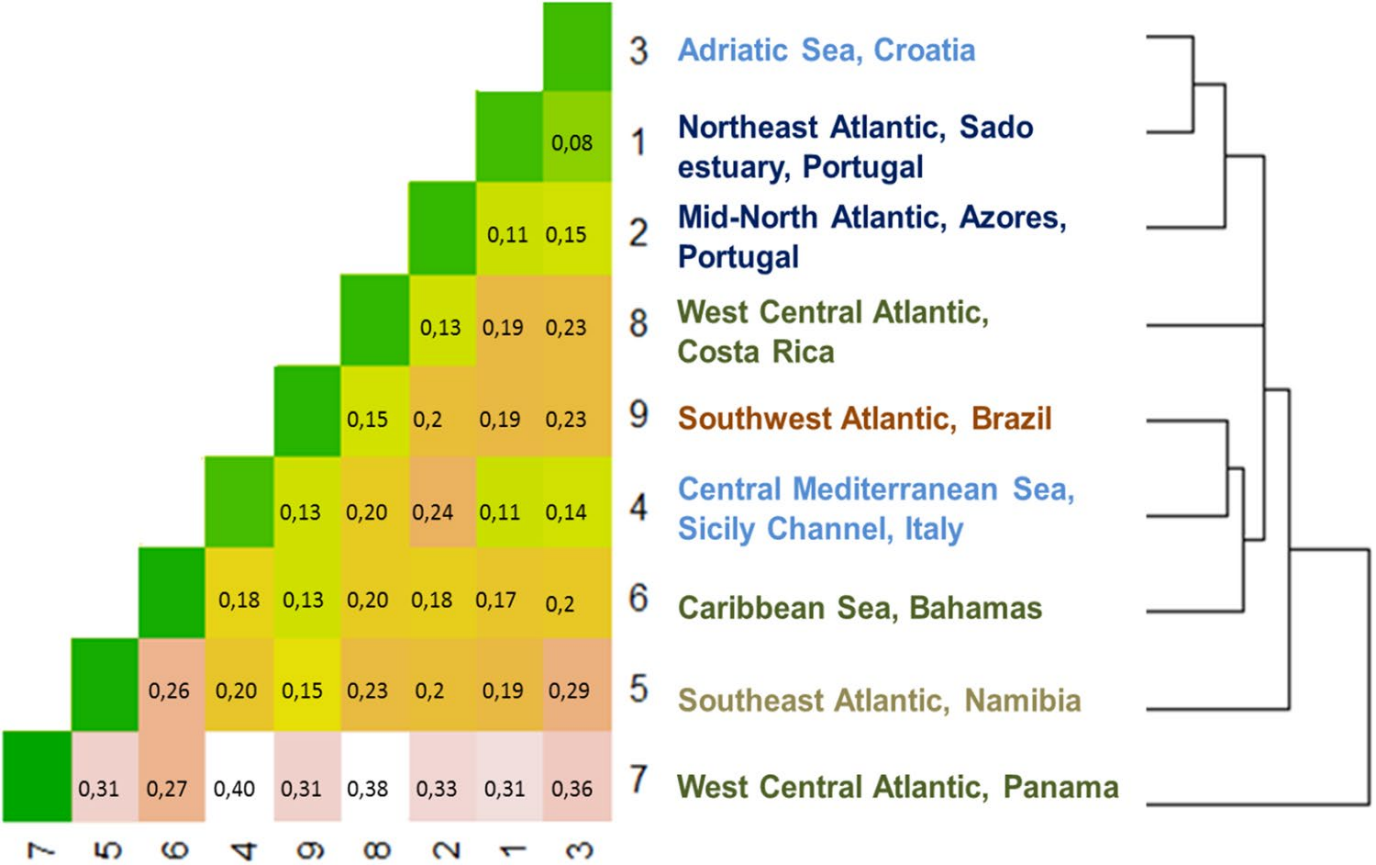
Acoustic repertoires’ similarity. Heatmap of dissimilarity values, with pairwise comparisons values and dendrogram of the hierarchical cluster analysis, made with R 3.5.0^45^, packages hcluster and ggplot2^46^.

Pairwise comparisons revealed different levels of repertoire similarity across populations (Fig. 1). Sado estuary and Adriatic Sea presented the highest repertoire similarity (dissimilarity, d = 0.08), while Panama had the most divergent acoustic repertoire, with dissimilarity values up to 0.4. Acoustic samples from Namibia were also distinct from the majority of other repertoires (d ≥ 0.2, except Brazil and Sado estuary). Azores had high similarity with two other northern hemisphere populations (Sado estuary: d = 0.11, Adriatic Sea, d = 0.15) but also with Costa Rica (d = 0.13). The highest dissimilarity value obtained for Costa Rica resulted from the comparison with Panama (d = 0.38). Sicily Channel had high similarity with other coastal northern hemisphere populations (Sado estuary: d = 0.11, Adriatic Sea: d = 0.14) but also with the coastal population of Brazil (d = 0.13). Additionally, Brazil presented high repertoire similarity with most of the southern hemisphere populations (Costa Rica: d = 0.13, Bahamas: d = 0.13 Namibia: d = 0.15).

## Discussion

This study compares acoustic signals emitted by common bottlenose dolphins from nine locations across the Atlantic Ocean and the Mediterranean Sea. Although unequal methodologies were used, in non-coincident time-frames, and while there are still numerous uncertainties regarding the infrageneric taxonomy of these delphinids, a broad discussion of the multi-regional repertoire within this cosmopolitan species is here attempted.

In the samples obtained from these nine populations, the repertoires included all the previously reported signal categories (whistles, burst-pulsed sounds, bray series elements and bangs), which we here classified in 24 nominal signal sub-types. Bottlenose dolphins are considered a highly vocal species, given both the diversity of calls and their often abundant emission rates^40^. Our results validate the assumption of a rich repertoire for this species, in line with values reported for other vocal groups such as birds and non-human primates^47,48^. Repertoires containing a large number of structurally and functionally distinct elements are often presented as a measure of complexity in communicative systems^49^. Our results reveal a wide diversity of calls, with structurally distinct elements, each with specific time–frequency features.

According to the Social Complexity Hypothesis for Communication^49^, animals that live in more complex social environments require more elaborate communication systems to regulate interactions and relations among group members. It is generally assumed that complex communication systems entail a larger number of signal types. Following these notions, one might expect that populations with larger group sizes would have more signal types. Repertoire sizes in this study varied from 13 to 19 sub-types but, interestingly, the largest repertoire was obtained from the smallest population (~ 30 individuals, Sado estuary, Portugal). In this stable resident community, other aspects of social complexity must be considered, such as often repeated interactions with many of the same individuals, in networks, over time. In primate societies with extensive affiliative relationships, animals use diverse vocal signal types to facilitate friendly interactions^50,51^. The large repertoire size in the Sado estuary could be related to the very high association indices presented by this population^52^. It should be noted that all Southern hemisphere populations had a repertoire size of 13 nominal call sub-types while all Northern hemisphere groups display slightly higher repertoire richness (an average of 17 sub-types). Our limited sample size, for some locations, precludes, at this stage, any further interpretation.

Despite the differences found in repertoire size, there were common signal types recorded in all nine locations. Whistles, creaks, squawks, variable rate click trains, bangs and squeaks were recorded at all sites, although for whistles and squeaks only specific sub-types occurred at all locations. The conspicuous occurrence of several pulsed signals (creaks, squawks, variable rate click trains, bangs) with specific food-related functions was expected, since feeding activities were, most likely, recorded at all sites. Although shifts in frequency and call rate may develop as a result of local habitat adaptations^53^, the occurrence of these signals seems universal and may result from selective pressures related with feeding efficiency. Regardless of location, habitat preferences, or designated subspecies, bottlenose dolphins sampled in this study produced variable rate click trains, creaks and squawks, described as pulsed calls emitted sequentially during feeding events^54^, and high-energy isolated pulses—bangs, which might play important roles in prey detection and startling^55,56^.

When it comes to social signals, such as whistles and bray series, acoustic divergence was noteworthy. While sinusoidal and convex whistles seem to be universal whistle types, other frequency modulated whistles were only recorded in some locations. Part of this variability may result from the unique contours of signature whistles, developed through vocal learning and used for long-term recognition of the individuals^57–61^. Here, whistles were grouped in general frequency modulation categories, regardless of the specific contour, and the weight of signatures whistles in each population repertoire was not accounted. Even so, it is interesting to verify that a few general whistle categories were only detected in specific repertoires. For example, low-frequency chirps were only recorded in the Bahamas and in Panama, locations that share a combination of ecological features absent at other sites: shallow-waters, high visibility, and coral reefs, which are known to have specific acoustic signatures. Frequency modulation of whistles is known to be influenced by the context of emission, namely the existence of different soundscape models and environmental constrains^41,53,62–64^. Thus, geographic variations in whistle emission, here documented, may reflect local adaptations to ambient noise backgrounds. Geographical proximity may also play an important role in the occurrence of shared vocal learned signals, as it has been portrayed for horizontal cultural transmission of humpback whales’ song^65,66^. In the current study, constant frequency whistles were shared by the two populations in the Mediterranean basin—Sicily Channel and Adriatic Sea, and also by the other closest population—Sado estuary, despite their differences in the ecological characteristics of the study-sites and the site-fidelity patterns of each group. Horizontal cultural transmission depends on social interactions, which are unlikely to occur between the resident populations of Adriatic Sea and Sado estuary; however, contact mediated by transient individuals that travel through the coastal waters of Mediterranean basin and North Atlantic is a possibility. Another relevant hypothesis is the occurrence of signal convergence/divergence for populations that form interspecific associations, namely in the Bahamas, with Atlantic spotted dolphins^67^ and Costa Rica, with Guiana dolphins^68^. In this respect, it would be interesting to look at the repertoires of those other, sympatric species.

Acoustic divergence was also observed for bray series elements, which presented high variability across populations. The nature of these information-rich vocalizations has several structural similarities with syllabic emissions in humpback whales and birds’ songs—sequential and timing aspects^39^. In birds and whales, vocal elements in songs have a strong social basis and the expression of geographic variants can be associated with the species’ vocal learning abilities—the individuals within a population learn specific vocal elements through a process of cultural transmission, horizontal or vertical^10,65^. Bray elements may have a similar social basis, especially considering that bottlenose dolphins are also vocal learners. However, for birds and whales, songs have been linked with sexual interactions^69,70^ whereas for dolphins brays have been associated with feeding events^71,72^, and social and aggressive behaviour^73^. One possibility is that brays might encode specific semantic content (prey-related or other) and may be produced only in certain social/cultural/environmental contexts, which would account for the geographic variability found in this study. Further investigation on the contextual use of bray series, at each location, is needed to elucidate the divergence patterns here presented.

Repertoire dissimilarity values express the acoustic divergence among populations, remarkably low in this multi-regional assessment. Although distinct ecotypes/ subspecies were sampled in this study, with unknown genetic relationships, acoustic repertoires’ similarity results do not reflect those differences—the Panama population had the most divergent acoustic repertoire, despite their ecotype similarity with most of the other populations, and *T. t. gephyreus* of Brazil presented high repertoire similarity with *T. t. truncatus* populations.

Divergence occurred only in social signals, suggesting an important role for cultural transmission. Likewise, acoustic similarity values did not mirror the geographic distance between groups or the site-fidelity patterns of the sampled populations, although interesting patterns emerged. The highest repertoire similarity values resulted from the comparison between the Sado estuary and Adriatic Sea populations—two resident groups that inhabit at shallow waters, with similar habitat features: muddy and sandy sediments with rocky reefs and seagrass meadows and high levels of ambient noise^52,53,74–76^.These habitat constrains could result in similar acoustic adaptation strategies to similar environmental challenges. In contrast, the lowest similarity value was obtained for closely located populations with distinct eco-ethological characteristics (Costa Rica—Panama). In Costa Rica, the dolphins show low site fidelity and the habitat extends to offshore waters, with little boat traffic^41^, while in Panama bottlenose dolphins show high degree of site fidelity^77^ and are exposed to high levels of anthropogenic noise due to the intensive vessel traffic^64^. These results strongly support the environmental adaptation hypothesis (for a review, see^78^)—when exposed to distinct environmental pressures, individuals would produce acoustic signals with time–frequency characteristics more adapted to specific environmental situations. In fact, soundscape might be the strongest selective pressure for acoustic emissions, as it affects vocalization transmission and reception, and ultimately survival. Individuals exposed to high levels of noise are known to alter emission rates and exhibit shifts in time–frequency parameters of acoustic elements^41,53,62,63^ Long-term exposure to noise may induce acoustic divergence/convergence that eventually may result in the presence/absence of acoustic units. Moreover, the diversity of eco-ethological contexts provides numerous communication challenges but also specific environmental acoustic stimuli. Given that bottlenose dolphins are proficient vocal learners, variability in acoustic ecology among populations may well contribute to geographic variation.

The existence of acoustically distinct populations, with variant social signals, could act as a significant interaction and reproduction barrier. Combining the analyses of genetic and acoustic structure could help to clarify the boundaries and relationships between neighboring groups, and shed light into vocal learning and cultural transmission in bottlenose dolphin societies.

Geographic variation and vocal identity are aspects of biodiversity, often undervalued, and the explicit identification of acoustically distinct groups may be relevant to future conservation strategies, as recognized by the Convention on Migratory Species (UNEP/CMS/Resolution 11.23, 2014).

## Methods

### Data collection

**Figure 2:**
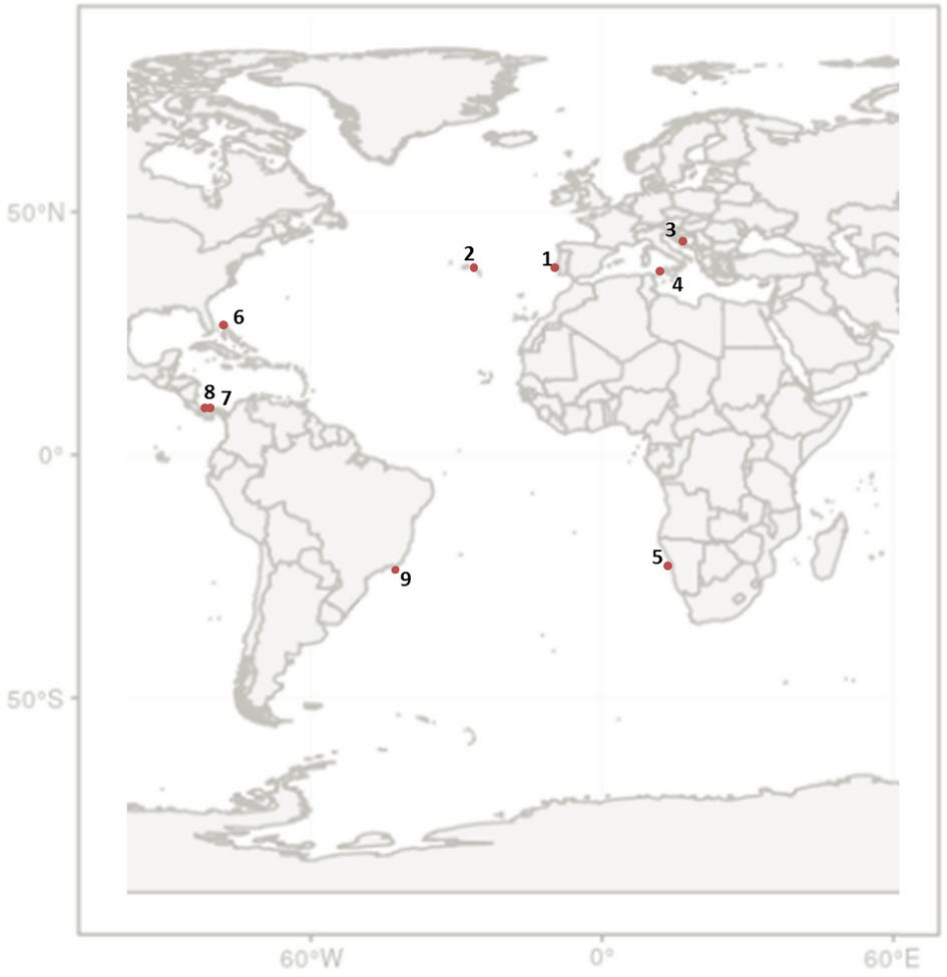
Geographic location of sampling sites. 1—Northeast Atlantic (Sado estuary, Portugal), 2—Mid-North Atlantic (Azores, Portugal), 3—Adriatic Sea (Cres-Lošinj, Croatia), 4—Central Mediterranean Sea (Sicily Channel, Italy), 5—Southeast Atlantic (Walvis Bay, Namibia), 6—Caribbean Sea (Bahamas), 7—West Central Atlantic (Bocas del Toro, Panama), 8—West Central Atlantic (Gandoca-Manzanillo, Costa Rica), 9—Southwest Atlantic (Rio de Janeiro coast, Brazil).

Bottlenose dolphins underwater vocalisations were recorded, from 2002 to 2016, at nine location (see Fig. 2): Northeast Atlantic (Sado estuary, Portugal), Mid-North Atlantic (Azores, Portugal), Adriatic Sea (Croatia), Central Mediterranean Sea (Sicily Channel, Italy), Southeast Atlantic (Namibia), Caribbean Sea (Bahamas), West Central Atlantic (Panama and Costa Rica), Southwest Atlantic (Brazil).

The sampling sites include coastal areas within the home-range of long-term studied resident populations, namely estuaries (Sado estuary, Portugal), bays (Walvis Bay, Namibia), and inshore archipelagic waters (Cres-Lošinj, Croatia, Bocas del Toro, Panama), but also nearshore sites regularly visited by groups with wider home-ranges that extend to offshore waters (Azores, Portugal, Sicily Channel, Italy, Bahamas, Rio de Janeiro, Brazil and Gandoca-Manzanillo, Costa Rica).

Coastal resident populations inhabit at shallow waters, with distinct habitat features: Sado estuary, Portugal and Adriatic Sea, Croatia sites include muddy and sandy sediments with rocky reefs and seagrass meadows; Walvis Bay, Namibia is a sandy-bottomed wetland; Bocas del Toro, Panama is characterized by clear waters and patchy substrate with mud, coral, seagrass, and mangroves. Rio de Janeiro, Brazil is a sandy-bottomed site under coastal and estuarine influence. Nearshore pelagic areas of Sicily Channel, with depths up to 500 m and rocky bottom, form an important ecological corridor that connects the western and the eastern Mediterranean Sea, whereas Azores is a mid-Atlantic hotspot of biodiversity, with deep-sea heterogeneous habitats that includes seamounts and hydrothermal vents. The Bahamas site, in the Great Bahama Bank, is a shallow area with high-visibility, sandy sea floor and scattered coral reefs, while Gandoca-Manzanillo, in the Caribbean coast of Costa Rica, is a shallow, muddy-bottomed bay, with estuarine influence, near a deep underwater canyon.

Acoustic data was collected with different recording systems, typically during dedicated boat-surveys, which included behavioral and photographic sampling. Specific methodological details for each site are described on Supplementary Table S1.

### Acoustic analyses

Sound recordings from all nine populations were inspected aurally, and visually using spectrograms plotted on Raven 1.4 (Cornell Lab of Ornithology, Ithaca, NY), with Hann windows of 512 points and a frequency resolution of 188 Hz and 50% overlap. Acoustic signals were rated according to the following signal quality index^79,80^: (i) poor—signal faint and hardly visible on the spectrogram, (ii) fair—signal visible and with a clear start/end on the spectrogram, (iii) good—signal well marked and with a clear start/end on the spectrogram. Signals rated as fair or good, and with no overlapping sounds, were selected for further analysis and classified as discrete vocal units.

Signal types were labeled as whistles, burst-pulsed sounds, bray series elements or bangs, according to previous descriptions^34,56,81–84^. Signal sub-types were defined using general time–frequency quantitative variation features, following^39,55,85^ (see Supplementary Table 2).

For each population, the repertoire composition was defined based on the occurrence of different signal types and sub-types.

### Repertoire similarity

The acoustic similarity among the repertoires of different populations was calculated using an index based on the degree of signal types shared. The similarity index is derived from Dice’s coefficient of association^86^ and takes into account differences in repertoire size:

$$Index\, of\,similarity=\frac{2{N}_{c}}{{R}_{1}+{R}_{2}}$$

where N_c_ is the total number of call types and sub-types shared, and R_1_ and R_2_ are the repertoire sizes (call types plus sub-types) of the two units.

As the similarity values are distance measures, we calculated its inverse to obtain the equivalent dissimilarity value (1—Index of similarity values) and computed a dissimilarity matrix. The dissimilarity matrix was used to perform a hierarchical cluster analysis, using the average linkage method. For visual comparison, a heat map with a dendrogram was plotted. Similarity analysis was performed using R Studio software, version 3.5.0, with hcluster and ggplot2 packages^45,46^.

## Supplementary Information

Supplementary Information.
