# Supplementary material for: Vocal universals and geographic variations in the acoustic repertoire of the common bottlenose dolphin
Source: Sci Rep. 2021 Jun 4;11:11847. doi: 10.1038/s41598-021-90710-9 (PMC8178411; doi:10.1038/s41598-021-90710-9)
Supplement: Supplementary file 1 — Supplementary Information. [file 41598_2021_90710_MOESM1_ESM.docx]

**Vocal universals and geographic variations in the acoustic repertoire of the common bottlenose dolphin**

Luís, A. R.^1, 2 *^, May-Collado, L. ^3, 4^ , Rako –Gospić, N. ^5^, Gridley, T. ^6^, Papale,. E ^7, 8^, Azevedo, A. ^9^, Silva, M. A. ^10^, Buscaino, G. ^7^, Herzing, D. ^11, 12^, & dos Santos, M. E. ^1, 2^

^1^MARE - Marine and Environmental Sciences Centre, ISPA - Instituto Universitário, Rua Jardim do Tabaco, 34, 1149-041 Lisboa, Portugal

^2^Projecto Delfim - Centro Português de Estudo dos Mamíferos Marinhos, Rua Jardim do Tabaco, 34, 1149-041 Lisboa, Portugal

^3^ Department of Biology, University of Vermont, Burlington, VT 05403.

^4^ Centro de Investigacion en Ciencias del Mar y Limnologia, Universidad de Costa Rica, San Jose Costa Rica

^5^ Blue World Institute of Marine Research and Conservation, Kaštel 24, 51551 Veli Lošinj, Croatia

^6^ Centre for Statistics in Ecology, Environment and Conservation, Department of Statistical Sciences, University of Cape Town, C/o Sea Search Research and Conservation NPC, Cape Town, South Africa

^7^ Institute for the Study of Antropogenic Impacts and Sustainability in the Marine Environment, National Research Council, Capo Granitola, Via del Mare 3, 91021 Torretta Granitola (TP), Italy

^8^ Department of Life Sciences and Systems Biology, University of Torino, Via Accademia Albertina 13, 10123 Torino, Italy

^9^ Laboratório de Mamíferos Aquáticos e Bioindicadores Profª Izabel Gurgel (MAQUA), Universidade do Estado do Rio de Janeiro – Brazil

^10^ OKEANOS & IMAR – Instituto do Mar, Universidade dos Açores, 9901-862 Horta, Portugal

^11^ Wild Dolphin Project, P.O. Box 8436 Jupiter FL 33468 USA

^12^ Department of Biological Sciences, Florida Atlantic University, Boca Raton, Fl 33431, USA

^*^ Correspondence: Ana Rita Luís, MARE – Marine and Environmental Research Centre, ISPA - Instituto Universitário, Rua Jardim do Tabaco, 34, Lisboa, P-1149-041, Portugal

*E-mail address*: aluis[@ispa.pt](mailto:manuel@ispa.pt) (Ana Rita Luís)

*Telephone number*: +351 964 715 842

Supplementary Table S1. Data collection

|  | **Time frame** | **Group size** | **Recording contexts** | **Hydrophone type** | **Functional bandwidth** | **Recorder type** | **Sampling rate** | **Additional details on study sites and acoustic recording methodology** |
| --- | --- | --- | --- | --- | --- | --- | --- | --- |
| **Northeast Atlantic**  **(Sado estuary, Portugal)** | Spring-Fall 2013, 2014 | 2-29 | Foraging/Milling, Surface Feeding, Social interactions, Travelling | Cetacean Research Technology, model C55 | 20 Hz to 100 kHz (sensitivity:−165 dB re 1V/ μPa) | Fostex FR-2 digital recorder | 192 kHz | *in* Luís et al., 2016,**^39^** |
| **Mid-North Atlantic**  **(Azores, Portugal)** | Summer 2002 | 14-66 | Foraging/Milling, Surface Feeding, Social interactions, Travelling | HTI-94-SSQ | 2 Hz to 30 kHz (ensitivity of−156 dB re 1 V/μPa) | Tascam DA-P1 | 48kHz | *in* Papale et al., 2014^44^ |
| **Adriatic Sea**  **(Croatia)** | Spring and Winter 2007, Winter 2008 | - | - | RESON TC 4032 | 5 Hz to120 kHz (sensivity: -170dB re 1V/ μPa) | Pioneer DC-88 DAT recorder | 44.1 kHz | *in* Rako et. al. 2013**^76^** |
|  | Spring and Summer 2016 | 2-29 | Foraging/Milling, Surface Feeding, Social interactions, Travelling, Fisheries interaction | RESON TC 4032 | 5 Hz to120 kHz (sensivity: -170dB re 1V/ μPa) | SOUNDDEVICES 702 | 192 kHz |  |
| **Central Mediterranean Sea**  **(Sicily Channel, Italy)** | Summer 2011, 2012, 2014 | 2-7 | Foraging/Milling, Surface Feeding, Social interactions, Travelling, Fisheries interaction, Resting | Bruel and Kjaer, model 8104 | 0.1 Hz –120 kHz (-205.6 dB re 1 V/ µPa +4/-12 dB) | Digital acquisition card Avisoft Ultra Sound Gate 416HB | 300 kHz | *in* Papale et al., 2015^87^ |
| **Southeast Atlantic (Namibia)** | Winter 2009 | 1-50 | Foraging/Milling, Surface Feeding, Social interactions, Travelling, Resting | High-Tec HTI-96-MIN | 2 Hz to 30 kHz (sensitivity: - 170dB re 1V/ μPa) | Edirol UA-25 sound card to PC (2009 recordings) | 96 kHz | *in* Gridley, T. et al., 2015^80^ |
|  | Summer 2011, 2012 |  |  |  |  | Zoom H4n digital recorder |  |  |
| **Caribbean Sea**  **(Bahamas)** | Summer 2002-2005, 2009 | 2-20 | Foraging/Milling, Social interactions | Custom-built omni-directional hydrophone | up to 300 kHz (sensitivity of –214 dB re 1V/μPa) | Underwater Dolphin Data Acquisition System (UDDAS) | 240 kHz | *in* Elliser & Herzing. 2015^88^, Hoffmann-Kuhnt, et al. 2016^89^ |
|  | Summer 2012, 2014 | 7-12 |  | RESON TC 4013 | 1Hz to 170kHz (sensitivity: -211dB ±3dB re 1V/µPa) | Zoom H4n digital recorder | 96 kHz |  |
| **West Central Atlantic**  **(Panama)** | Summer 2004, 2007, 2008, 2012 | 2-10 | Foraging/Milling, Social interactions, Travelling | RESON TC 4033 | 1 Hz to 140 kHz (sensitivity: - 203 dB re 1V/ μPa) | Digital acquisition card Avisoft Ultra Sound Gate 116 | 384 kHz | *in* May-Collado & Quiñones-Lebrón, 2014^64^ |
| **West Central Atlantic**  **(Costa Rica)** | Summer 2004, 2007 | - | Foraging/Milling, Social interactions, Travelling | RESON TC 4033 | 1 Hz to 140 kHz (sensitivity: - 203 dB re 1 V=1 μPa) | Digital acquisition card Avisoft Ultra Sound Gate 116 | 384-500 kHz | *in* May-Collado, L.. et al., 2010^80^ |
| **Southwest Atlantic**  **(Brazil)** | Spring 2014 | - | - | High-Tec HTI-96-MIN | 2 Hz to 30 kHz (sensitivity: - 170dB re 1V/μPa) | Fostex FR-2 digital recorder | 96 kHz | *in* Lima, S. et al., 2016^90^ |
|  | Spring 2015 | 20 | Travelling | Cetacean Research Technology, model c54 XRS | 9 Hz a 100 kHz (sensitivity: -165 dB re: 1 V/µPa) |  |  |  |

Supplementary Table S2. Description of the general acoustic categories

| Signal type | Signal Sub-types | Description | Example |
| --- | --- | --- | --- |
| Whistles |  | **Tonal, narrow-band, modulated signals** |  |
|  | Upsweep | Frequency increasing over time, no inflection points | 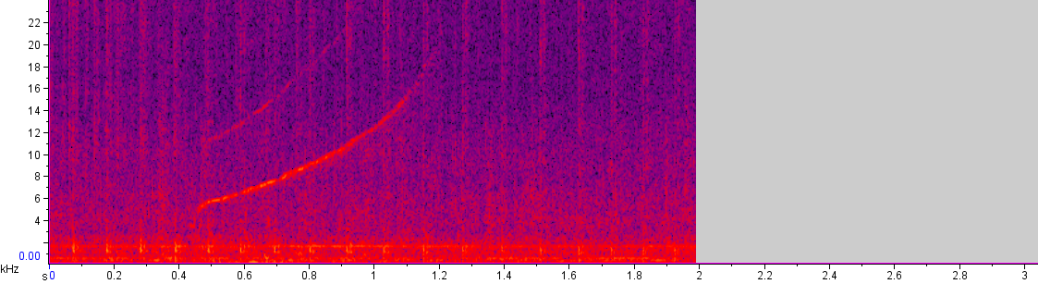 |
|  | Downsweep | Frequency decreasing over time, no inflection points | 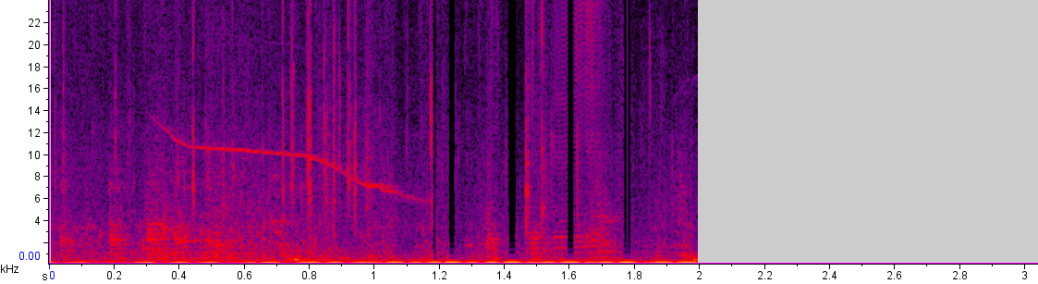 |
|  | Convex | Frequency initially increasing followed by an inflection point and an ending portion decreasing | 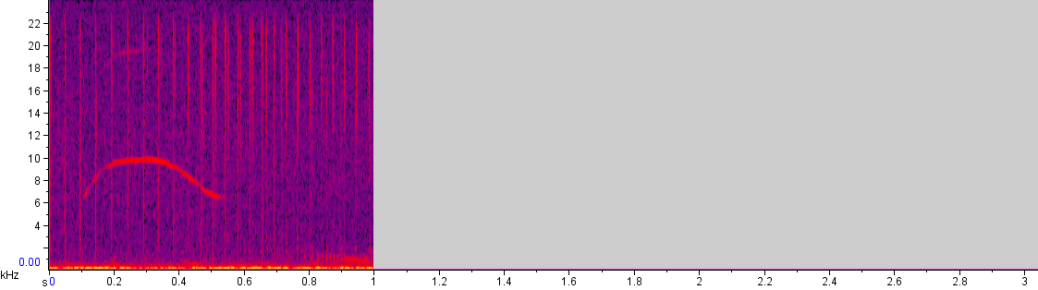 |
|  | Concave | Frequency initially decreasing followed by an inflection point and an ending portion increasing | 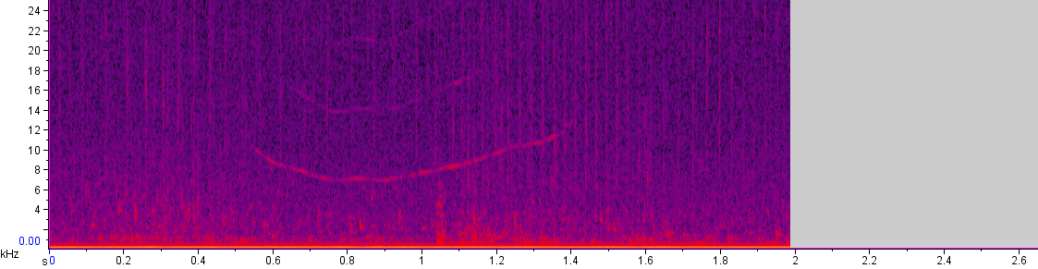 |
|  | Sinusoidal | Multiple inflection points | 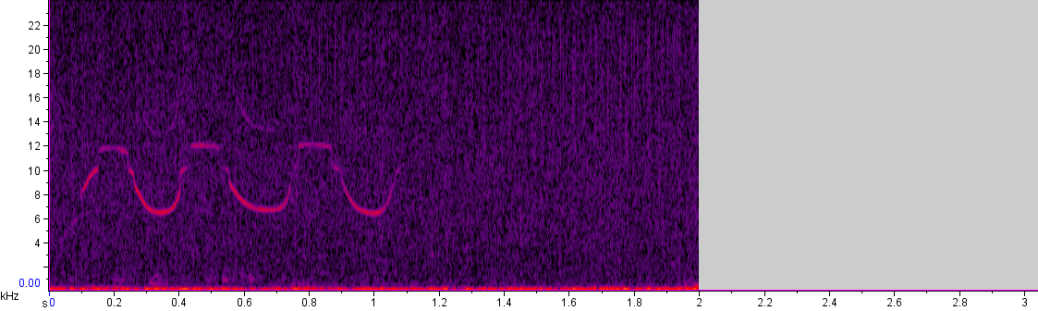 |
|  | Constant | Frequency variation less than 25%, no inflection points | 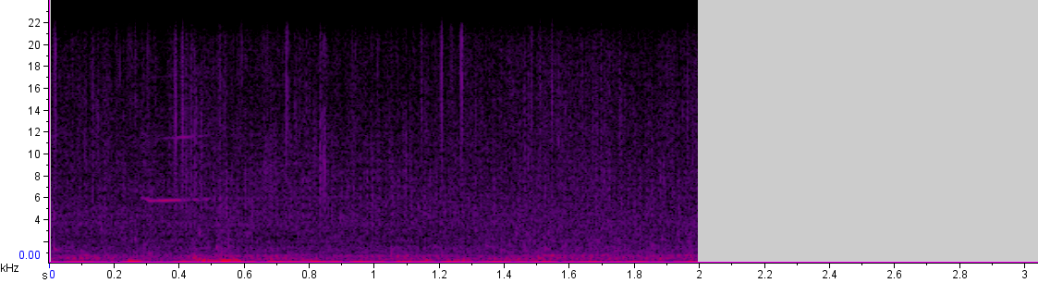 |

| BP sounds |  | Packets of high repetition, broadband pulses with inter-click-intervals below 10 ms |  |
| --- | --- | --- | --- |
|  | Creaks (or buzzes) | Long burst-pulse (>2 ms), aurally similar to a creaking door | 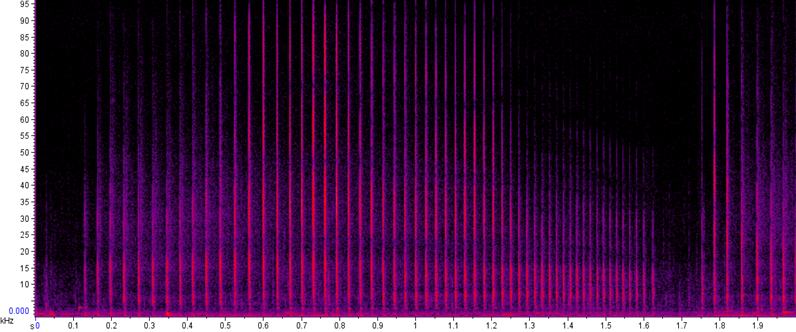 |
|  | Squawks | Long burst-pulse (>2 ms), with higher repetition rate than “Creaks”, reminiscent of a crying baby | 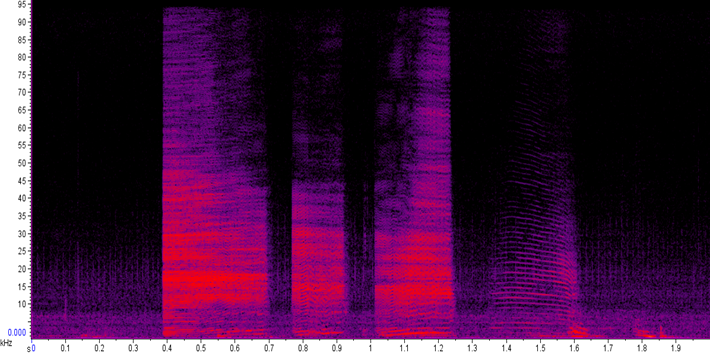 |
|  | Short burst-pulse  (S-BP) | Short burst-pulse (<2 ms), aurally similar to a buzzing bee but brief | 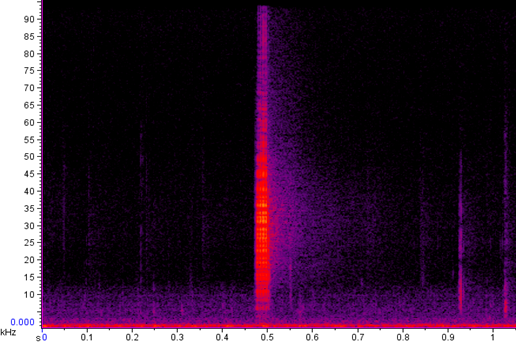 |
|  | Variable rate click train (VRCT) | Long, graded click series that include continuous creaks and squawks | 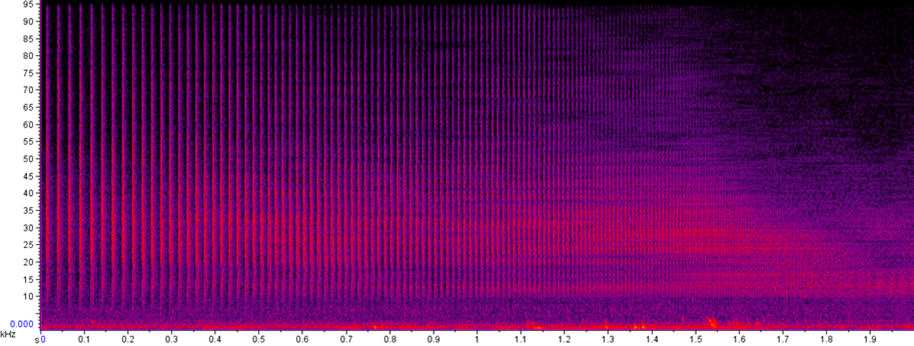 |

| Bray series elements |  | Rhythmic sequences up to 30s in length, composed by short duration pulsed or tonal signals |  |
| --- | --- | --- | --- |
|  | Gulps (or LFN) | Low-frequency narrow-band sounds | 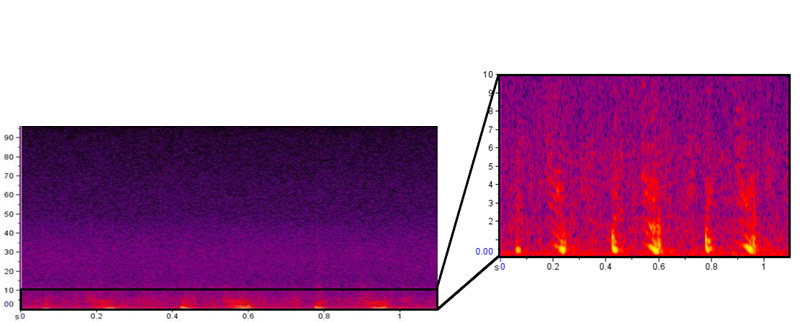 |
|  |  |  |  |
|  |  |  |  |
|  | Grunts | Broadband burst pulses, with strong emphasis in the lower frequencies | 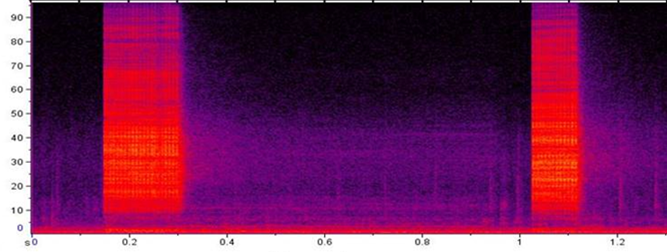 |
|  | SC-squeaks | Short duration (<2 ms), high repetition rate harmonic structure with convex modulation | 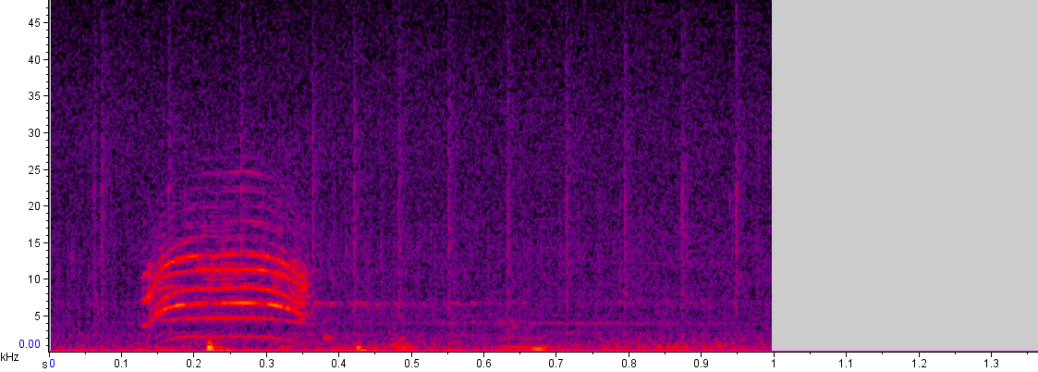 |
|  | VSC-squeaks | Very short duration (<1 ms), high repetition rate harmonic structure with convex modulation | 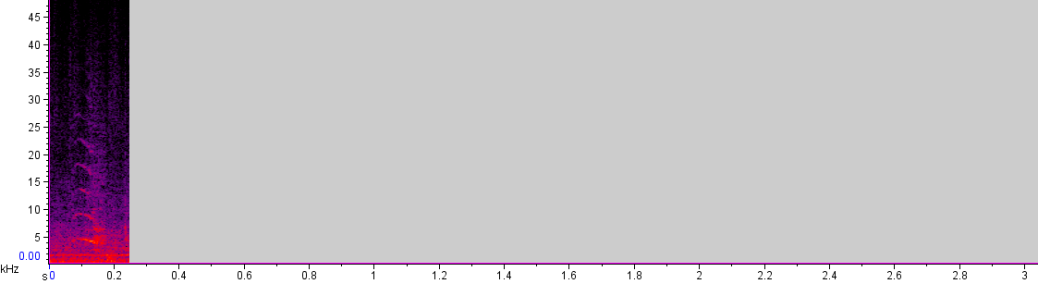 |
|  | Up-squeaks | Very short duration (<1 ms), high repetition rate harmonic structure with upsweep modulation | 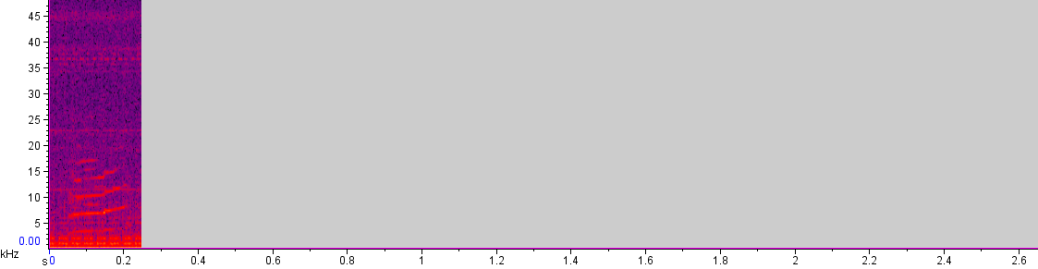 |
|  | D-squeaks | Very short duration (<1 ms), high repetition rate harmonic structure with downsweep modulation | 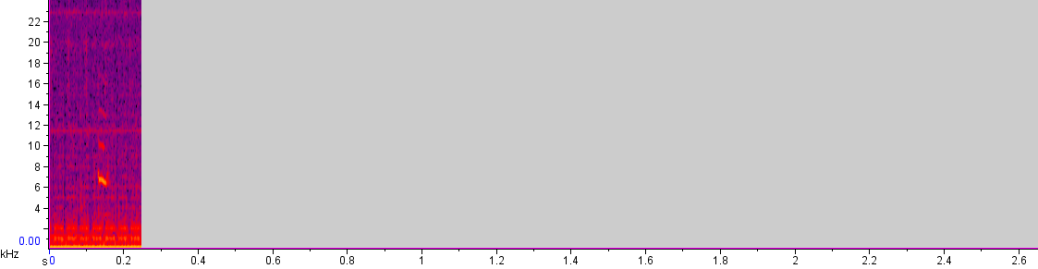 |
|  | LD-squeaks | Short duration (<2 ms), high repetition rate harmonic structure with downsweep modulation | 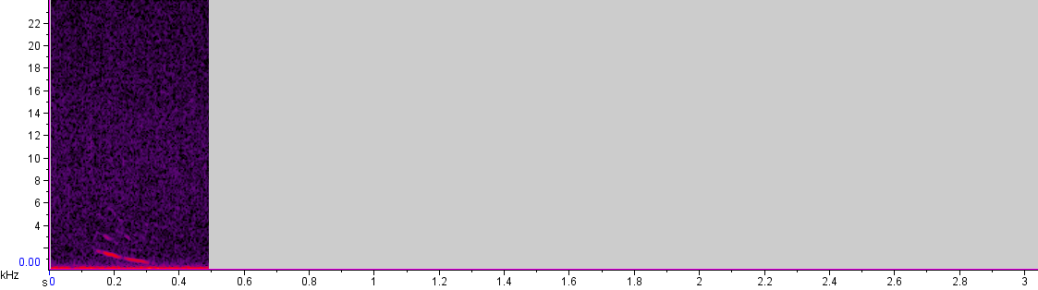 |
|  | Un-squeaks | Short duration (<2 ms), high repetition rate harmonic structure with undefined modulation | 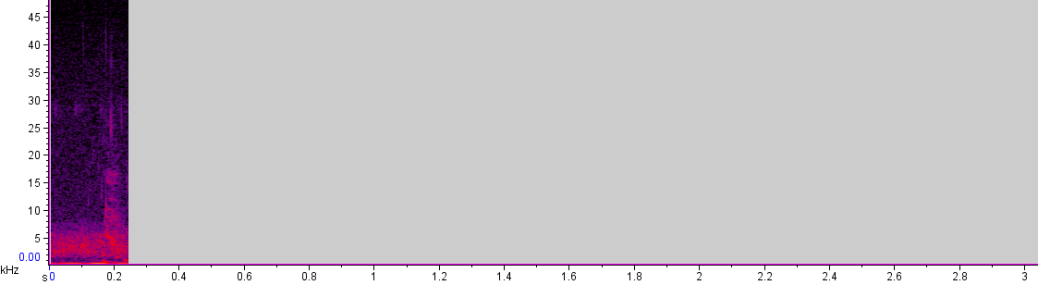 |
|  | Cc-squeaks | Short duration (<2 ms), high repetition rate harmonic structure with concave modulation | 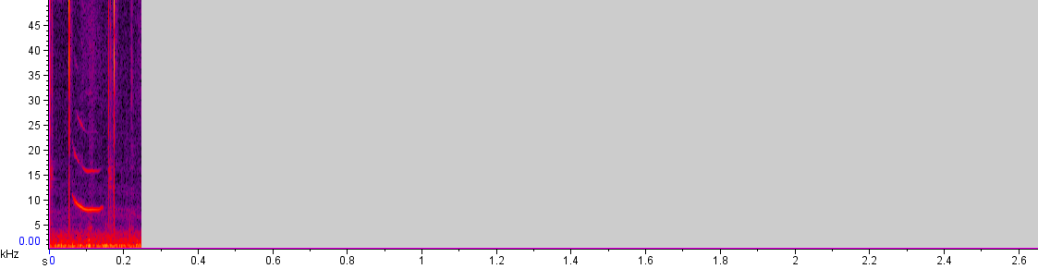 |
|  | Sin-squeaks | Short duration (<2 ms), high repetition rate harmonic structure with sinusoidal modulation | 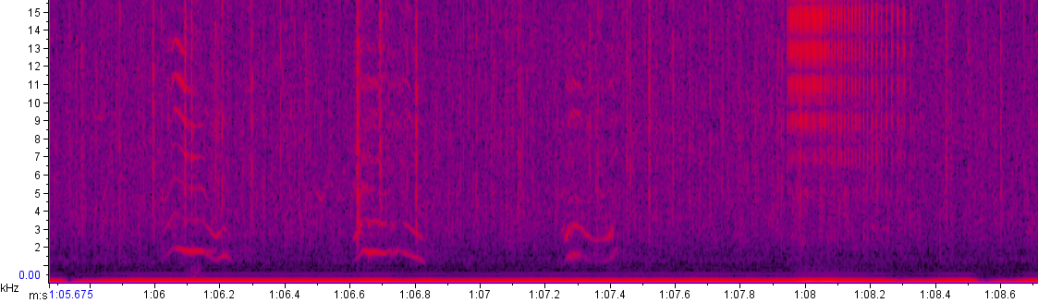 |
| Bangs |  | **Isolated, high-energy, broadband pulses** | 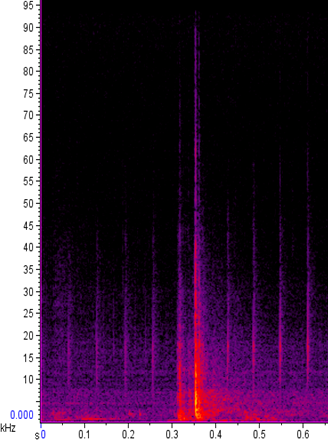 |
